# Identification and validation of autophagy‐related genes in exogenous sepsis‐induced acute respiratory distress syndrome

**DOI:** 10.1002/iid3.691

**Published:** 2022-09-23

**Authors:** Yongpeng Xie, Wenxia Hu, Xiaobin Chen, Panpan Ren, Chongchong Ye, Yanli Wang, Jiye Luo, Xiaomin Li

**Affiliations:** ^1^ Department of Emergency and Critical Care Medicine, Lianyungang Clinical College of Nanjing Medical University The First People's Hospital of Lianyungang Lianyungang Jiangsu China; ^2^ The Institute of Emergency Medicine of Lianyungang Lianyungang Jiangsu China

**Keywords:** acute respiratory distress syndrome, autophagy, hub gene, sepsis, transcriptomics

## Abstract

**Objective:**

To analyze the differential expression of autophagy‐related genes of sepsis‐induced acute respiratory distress syndrome (ARDS) as potential markers for early diagnosis.

**Methods:**

Male Sprague–Dawley rats (aged 8 weeks) were selected and randomly divided into sepsis‐induced ARDS group (*n* = 6) and a normal control group (*n* = 6). Lung tissue samples were collected for high‐throughput sequencing using Illumina HiSeq sequencing platform in the paired‐end sequencing mode. Differentially expressed genes (DEGs) were screened by DESeq. 2 software [|log2FC | ≥1 and *p* < .05] and autophagy‐related genes were identified using Mouse Genome Informatics. Co‐expressed autophagy‐related DEGs from these two datasets were filtered by construction of a Venn diagram. Gene ontology (GO) and Kyoto Encyclopedia of Genes and Genomes (KEGG) pathway enrichment analyses were performed on these autophagy‐related DEGs and a protein interaction network was constructed using STRING and Cytoscape software to identify hub genes, which were verified by real‐time quantitative polymerase chain reaction (qRT‐PCR).

**Results:**

A total of 42 autophagy‐related DEGs (26 upregulated genes and 16 downregulated genes) were identified. The GO and KEGG pathway analyses showed enrichment in 969 biological processes (BPs), three cellular components (CCs), eight molecular functions (MFs) and 27 signaling pathways. The protein interaction (PPI) network revealed 42 node proteins and 75 interacting edges, with an average node degree of 3.52, and an average local clustering coefficient of 0.509. Among the top 10 hub genes with the RNA‐Seq, six hub genes (Stat3, Il10, Ifng, Hmox1, Hif1a, and Nod2) were validated by qRT‐PCR (all *p* < .05).

**Conclusion:**

42 potential autophagy‐related genes associated with sepsis‐induced ARDS lung injury were identified and six hub genes (Stat3, Il10, Ifng, Hmox1, Hif1a, and Nod2) may affect the development of ARDS by regulating autophagy. These results expanded our understanding of ARDS and might be useful in treatment of exogenous sepsis‐induced ARDS.

## Graphical abstract

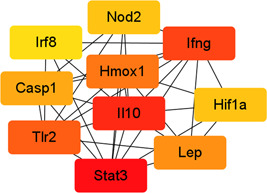

## 1 INTRODUCTION

Sepsis is still a serious medical condition, with a global incidence of around 437/100,000 of the population, and an annual death toll of about 5.3 million.^1^ The 2021 international guidelines for the management of septic shock defined sepsis as life‐threatening organ dysfunction resulting from a dysregulated response to infection.^2^ The lung is one of the earliest and most easily affected organs among the various organ functions of the human body.^3^ The clinical manifestations are mainly acute respiratory distress syndrome (ARDS), which is one of the most common acute and serious illnesses in the field of critical medicine, with a fatality rate of over 40%.^4^ Although significant progress has been made in the understanding and treatment of ARDS pathophysiology in the past 50 years, the mechanism of acute lung injury caused by sepsis is still not fully understood, and there are still no specific biomarkers and effective prevention and treatment targets.^5^ Studies^6^, ^7^ have shown that ARDS lung injury is characterized by inflammatory processes demonstrated as loss of function of the pulmonary capillary endothelial and alveolar epithelial cells. Autophagy is an intracellular digestion system that work as an inducible adaptive response to lung injury which is a resultant of exposure to various stress agents like hypoxia, sepsis, ischemia‐reperfusion, and xenobiotics which may be manifested as acute lung injury (ALI), ARDS, asthma, ventilator‐induced lung injury (VILI), pulmonary fibrosis.^8^ Numerous regulators like LC3B‐II, Beclin 1, p62, HIF1/BNIP3, and mTOR may play pivotal role in autophagy induction during lung injury for progression/inhibition of the disease state.^9^ However, the mechanism of autophagy in septic ARDS lung injury is still unclear. In this study, we used RNA‐seq technology to screen the autophagy‐related genes and pathways involved in the process of lung injury caused by exogenous factors in sepsis‐induced ARDS. To explore the critical autophagic mediators and their potential cross talk with the lung injury thereby bringing to limelight the possible therapeutic interventions.

## 2 MATERIALS AND METHODS

### 2.1 Experimental animals and groups

This study was approved by the Medical Ethics Committee of Lianyungang Clinical College of Nanjing Medical University (China), and all animal studies were conducted in strict accordance with the requirements of the animal experimentation ethics committee (KY20200311001). Twelve healthy male Sprague–Dawley (SD) rats (aged 6–8 weeks, weighing 230–250 g) were allocated According to the random number table method, they were divided into control (N group) and experimental (LPS group) groups using the random number table method, with six animals in each group. In the LPS group, a sepsis‐induced ARDS lung injury model was established by intraperitoneal injection of lipopolysaccharide (LPS; Sigma‐Aldrich; 15mg/kg).

### 2.2 Pathological evaluation of lung tissue

After 36 h, all rats were anesthetized by intraperitoneal injection of xylazine (Sigma‐Aldrich; 8mg/kg) and ketamine (Hengrui; 80mg/kg) before the rats were killed by cardiac puncture and bloodletting, and specimens were collected for analysis. For each rat, the right upper lobe of the lung was collected, dehydrated with alcohol, embedded in paraffin, and sectioned. All the pathological specimens were stained with hematoxylin‐eosin and observed under a light microscope (Olympus) at low, medium, and high magnification.

### 2.3 Total RNA extraction from lung tissue

Total RNA from the left lung of rats was extracted using the column method, and the purity and integrity of the RNA samples were detected using a Nanodrop one ultra‐micro spectrophotometer and an Agilent 2100 bioanalyzer, respectively, to ensure that the RNA quality met the requirements for subsequent sequencing.

### 2.4 Transcriptome data processing and acquisition of autophagy‐related gene datasets

High‐throughput sequencing of multiple RNA samples was conducted using the Illumina HiSeq sequencing platform in the paired‐end sequencing mode. Skewer software (v0.2.2) was used to dynamically remove adapter sequence fragments and low‐quality fragments from the 3′‐end of the sequencing data, and FastQC software (v0.11.5) was used to perform pre‐processing data quality control. For each sample, STAR software (2.5.3a) was used to align the pre‐processed sequence with the reference genome sequence of the sequenced species, and RSEQC (v2.6.4) was used for comparative analysis. The FKPM (fragments per kilobase of exon model per million mapped fragments) method was used to calculate the gene expression, and the differentially expressed genes (DEGs) were screened using |log2FC | >1 and *p* < .05 as the criteria. A total of 490 autophagy‐related genes were obtained from The Mouse Genome Informatics Database when we entered keywords “autophagy,” Filtered by feature types of gene/lncRNA gene/noncoding RNA gene/protein coding gene and pseudogene (http://www.informatics.jax.org/).

### 2.5 Analysis of differentially expressed autophagy‐related genes

Venn diagrams were constructed to filter co‐expressed autophagy‐related DEGs from the N and LPS group datasets. DEGs were screened using an adjusted *p* < .05 and |log2FC | >1.0 as the criteria. Heatmaps was generated using the “heatmap” package of R software.

### 2.6 Gene ontology (GO) and Kyoto Encyclopedia of Genes and Genomes (KEGG) pathway enrichment analysis of autophagy‐related genes

GO function enrichment analysis and KEGG pathway enrichment analysis were performed for functional annotation and classification of co‐expressed autophagy‐related DEGs.

### 2.7 Protein–protein interaction (PPI) analysis and correlation analysis of autophagy‐related DEGs

For PPI analysis, the co‐expressed autophagy‐related DEGs were imported into the online database STRING, and the proteins with a comprehensive score >0.7 in the PPI network graph were regarded as statistically significant. Spearman correlation analysis of the differentially expressed autophagy‐related genes was performed using the “corrplot” package of R software. The top 10 most significant key genes were screened using the cytoHubba plug‐in of Cytoscape (version 3.9.1).

### 2.8 RNA extraction and real‐time quantitative polymerase chain reaction (qRT‐PCR) analysis

The top 10 hub genes screened out were verified by qRT‐PCR. Total RNA was extracted from lung tissue samples isolated from 20 sepsis‐induced ARDS rats and five normal control rats. The cDNA was generated by reverse transcription of the extracted RNA using the PrimeScript RT Master Mix kit (TaKaRa, Dalian). The qRT‐PCR analysis of autophagy‐related DEG expression in lung tissue was performed using the Ultra SYBR Mixture with the following reaction conditions: 95°C for 10 min, followed by 45 cycles of 95°C for 15 s, and 60°C for 60 s. Actin was as an internal reference gene for calculation of the relative expression of mRNA using a 2^−ΔΔCT^ method. The gene primer sequences used in this study are shown in Table 1.

**Table 1:** Primer sequences

Primer name	Forward primer (5′‐>3′)	Reverse primer (5′‐>3′)	Primer base pair
Nod2	GCACTTCCATTCCATCCC	CACCCTGCAAAACGTCAA	172
Ifng	AACTGGCAAAAGGACGGT	TCAGGTGCGATTCGATGA	130
Stat3	CCATCCTAAGCACAAAGC	AGTGAAAGTGACCCCTCC	80
Il10	GACCCACATGCTCCGAGA	TGGCAACCCAAGTAACCC	132
Tlr2	TACCTGTGTGATTCTCCG	TTTCTTGGGCTTCCTCTT	216
Hmox1	CCAGCCACACAGCACTAC	CATGCGAGCACGATAGAG	234
Lep	CCCCATTCTGAGTTTGTC	GGAGGTCTCGCAGGTTCT	122
Casp1	TGGAGAGAAACAAGGAGT	AGAGCAGAAAGCAATAAA	136
Hif1a	TCCCATACAAGGCAGCA	GAAACCCCACAGACAACAA	140
Irf8	GGCATCTCACCCTTTCCT	GCATACCCTTTCCCTGGT	117
Actin	CCCATCTATGAGGGTTACGC	TTTAATGTCACGCACGATTTC	150

## 3 RESULTS

### 3.1 Pathological changes and analysis of autophagy‐related DEGs

**Figure 1:**
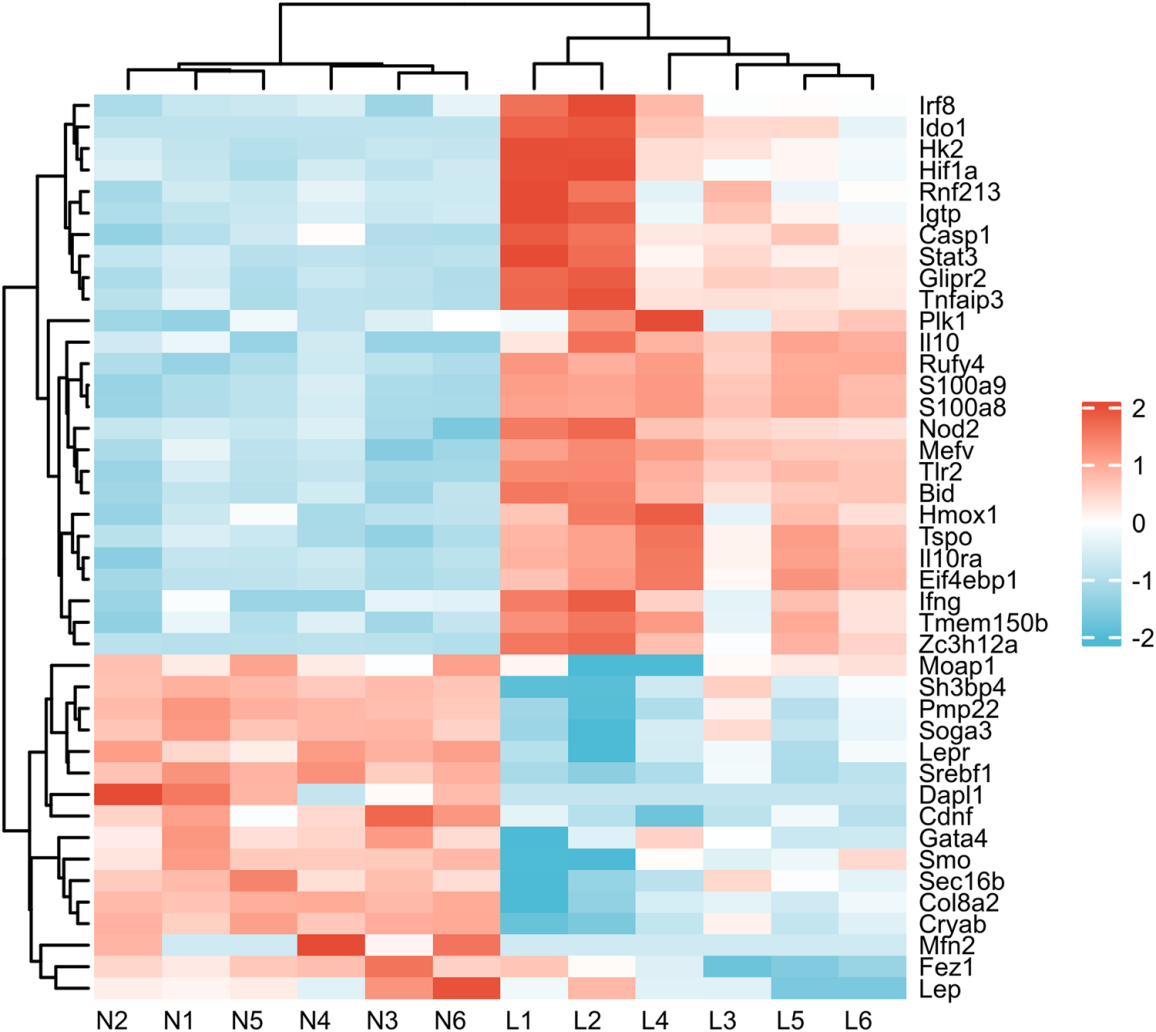
Heatmap of the 42 differentially expressed autophagy‐related genes in ARDS and normal samples. 26 upregulated genes and 16 downregulated using the adjusted *p* < .05 and absolute fold‐change value >1 as the criteria including. ARDS, acute respiratory distress syndrome.

At 36 h after establishment of the sepsis‐induced ARDS lung injury model, the lung tissue in the LPS group showed more serious pathological damage, manifested as diffuse alveolar damage, with intra‐plasmic congestion and edema, inflammatory cell infiltration, atelectasis, and hyaline membrane formation. In contrast, no obvious pathological changes in lung tissue were in the control group. Based on |log2FC | ≥ 1 and *p* < .05 as the criteria, a total number of 3541 DEGs were screened by comparison of the data obtained from the control and LPS groups using DESeq. 2 software. A total of 490 autophagy‐related genes were obtained from The Mouse Genome Informatics Database. By construction of Venn diagrams, we identified 42 autophagy‐related DEGs (26 upregulated genes and 16 downregulated) using the adjusted *p* < .05 and absolute fold‐change value >1 as the criteria including (Figure 1).

### 3.2 GO and KEGG enrichment analysis of the differentially expressed **autophagy‐related genes**

**Figure 2:**
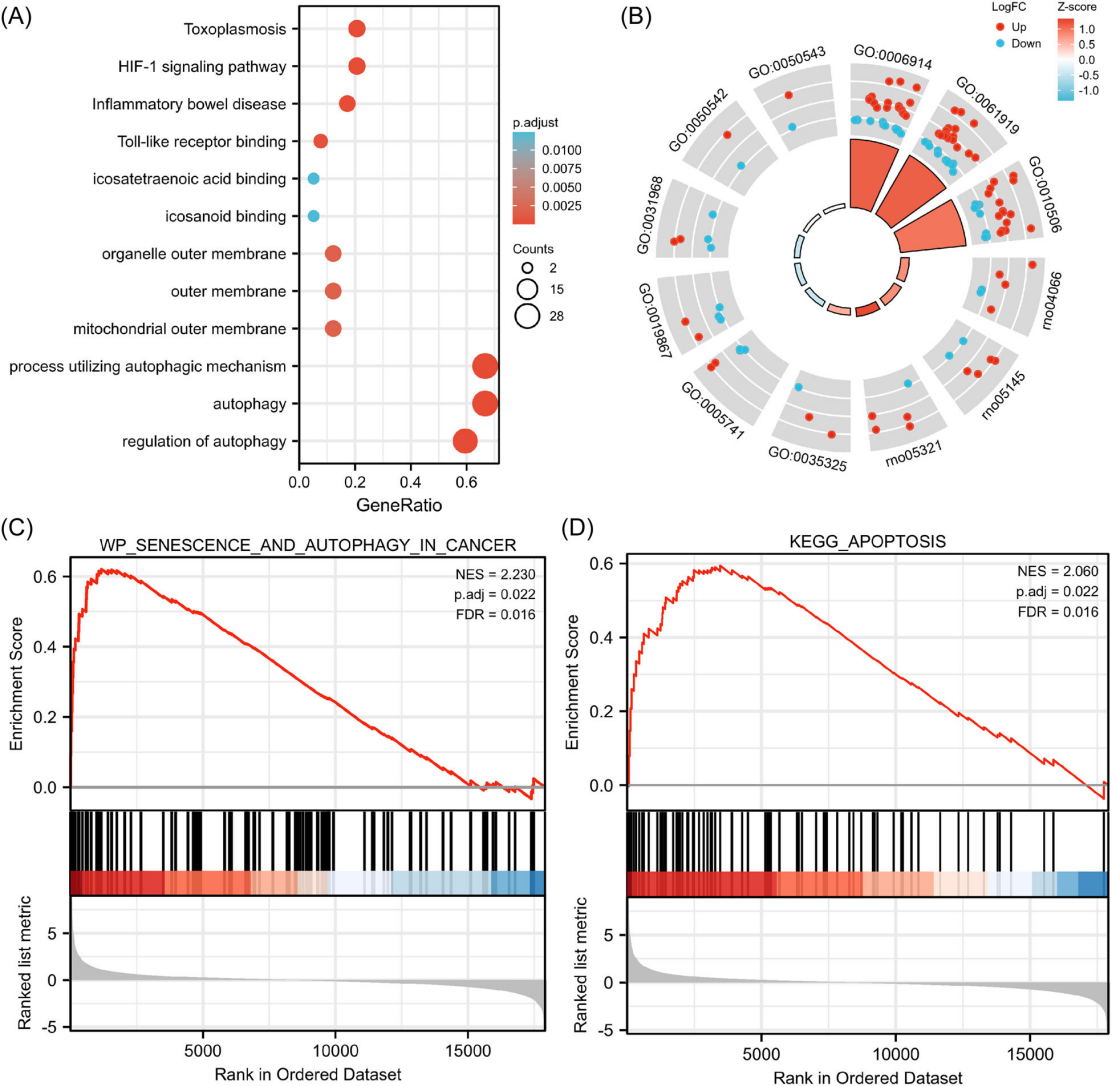
GO/KEGG enrichment analysis of DEGs and GSEA of all detected genes. (A) Bubble plot showing the distribution of DEGs in the molecular function (MF), biological process (BP), and cellular components (CC) categories. (B) Circle plot showing the distribution of DEGs in the different GO KEGG categories combined with logFC. (C) and (D) shown the enrichment of gene sets in autophagy and apoptosis, respectively. DEGs, differentially expressed genes; GO, gene ontology; GSEA, Gene Set Enrichment analysis; KEGG, Kyoto Encyclopedia of Genes and Genomes.

The GO function analysis of 42 autophagy‐related DEGs showed enrichment in 969 biological processes (BPs), three cellular components (CCs), and eight molecular functions (MFs). The biological processes involved in autophagy‐related DEGs were mainly autophagy, process utilizing autophagic mechanism and regulation of autophagy. In the CC category, DEGs were mainly enriched in mitochondrial outer membrane, outer membrane and organelle outer membrane. In the MF category, DEGs were significantly enriched in Toll‐like receptor binding, eicosanoid binding, and eicosatetraenoic acid binding. In the biological processes category, DEGs were significantly involved in the HIF‐1 signaling pathway, JAK‐STAT signaling pathway and toxoplasmosis (Table 2). The KEGG pathway analysis showed that 27 signaling pathways that were significantly enriched in the septic ARDS lung injury group (Figure 2A,B). GSEA analysis showed that both autophagy‐ and apoptosis‐related genes were upregulated in the ARDS lung injury group (Figure 2C,D) and had significant biological functions.

**Table 2:** GO and KEGG enrichment of autophagy‐related DEGs

Ontology	ID	Description	Gene ratio	Bg ratio	*p* value	*p*. adjust	*q* value
BP	GO:0006914	Autophagy	28/42	385/17,962	2.81e−37	3.61e−34	1.68e−34
BP	GO:0061919	Process utilizing autophagic mechanism	28/42	385/17,962	2.81e−37	3.61e−34	1.68e−34
BP	GO:0010506	Regulation of autophagy	25/42	255/17,962	3.96e−36	3.40e−33	1.58e−33
CC	GO:0005741	Mitochondrial outer membrane	5/41	151/18,446	2.03e−05	.002	.001
CC	GO:0019867	Outer membrane	5/41	174/18,446	4.01e−05	.002	.001
CC	GO:0031968	Organelle outer membrane	5/41	174/18,446	4.01e−05	.002	.001
MF	GO:0035325	Toll‐like receptor binding	3/39	13/16,882	3.21e−06	5.17e−04	3.58e−04
MF	GO:0050542	Icosanoid binding	2/39	10/16,882	2.31e−04	.012	.009
MF	GO:0050543	Icosatetraenoic acid binding	2/39	10/16,882	2.31e−04	.012	.009
KEGG	rno04066	HIF‐1 signaling pathway	6/29	120/9437	1.39e−06	7.18e−05	4.76e−05
KEGG	rno04630	JAK‐STAT signaling pathway	6/29	162/9437	7.99e−06	2.48e−04	1.64e−04
KEGG	rno05145	Toxoplasmosis	6/29	122/9437	1.54e−06	7.18e−05	4.76e−05

Abbreviations: DEGs, differentially expressed genes; GO, gene ontology; KEGG, Kyoto Encyclopedia of Genes and Genomes.

### 3.3 PPI interaction analysis

**Figure 3:**
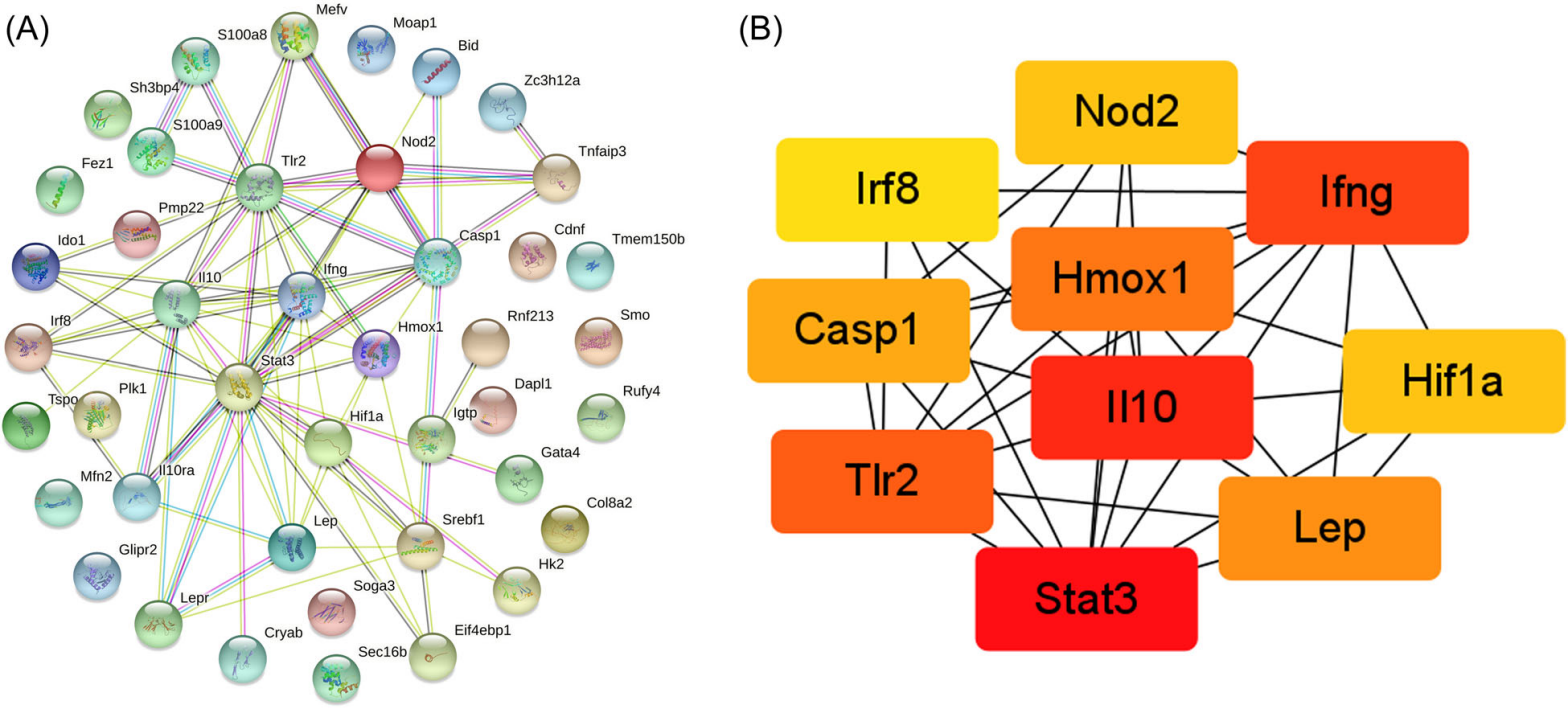
(A) Protein‐protein interaction (PPI) network of autophagy‐related differentially expressed genes (DEGs). The nodes indicated proteins and the letters indicated corresponding gene symbols. (B) A total of 10 hub genes were identifified using Cytohubba plugin in Cytoscape, with darker color representing higher score.

A PPI network diagram of the 42 autophagy‐related DEGs was shown in Figure 3A. Among the 42 node proteins, 75 interacting edges (the expected number of edges was 123) were screened, with an average node degree of 3.52, an average local clustering coefficient of 0.509, and enrichment *p* < .001. Using the Cytoscape plug‐in cytoHubba, the top 10 hub genes with degree values >10 were: Stat3, Il10, Ifng, Tlr2, Hmox1, Lep, Casp1, Hif1a, Nod2, and Irf8 (Figure 3B).

### 3.4 Validation of the differentially expressed autophagy‐related genes in ARDS rats by qRT‐PCR

**Figure 4:**
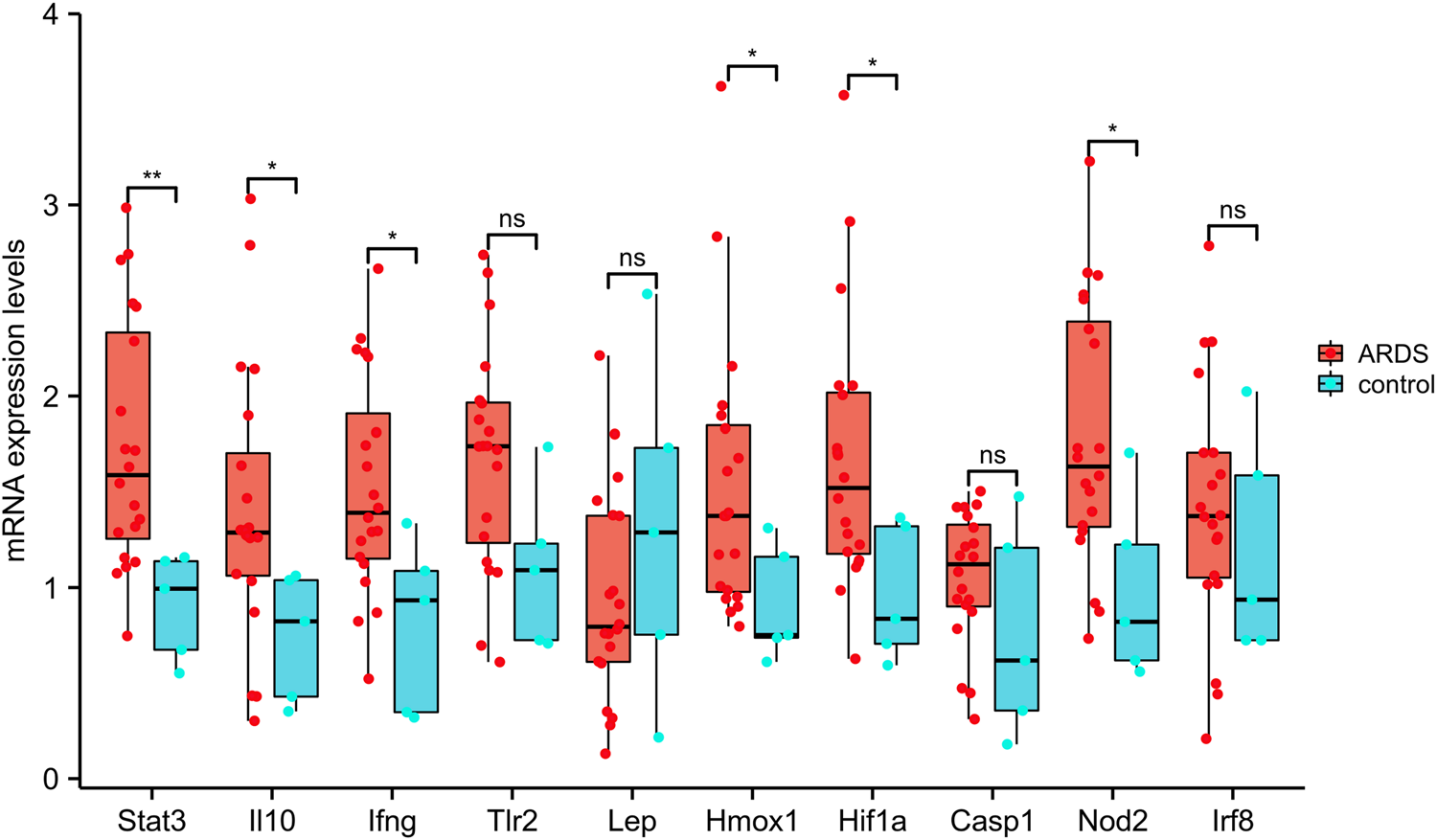
Validation of the top 10 hub differentially expressed autophagy‐related genes in rat lung tissue by qRT‐PCR. qRT‐PCR, real‐time quantitative polymerase chain reaction.

The top 10 hub autophagy‐related DEGs identified by RNA‐seq were validated by qRT‐PCR analysis of the lung tissue samples of 20 ARDS rats and five control rats. The results showed that the trend in the expression of six key hub genes (Stat3, Il10, Ifng, Hmox1, Hif1a, and Nod2) was consistent with the RNA‐seq results (all *p* < .05), while there was no significant difference in the expression of the other four genes between the two groups (Figure 4).

## 4 DISCUSSION

Research has revealed distinct differences in clinical characteristics, molecular mechanisms and treatment responses among the ARDS subtypes.^10^ Clinical studies have shown that patients with sepsis‐induced ARDS phenotype are more severely ill than non‐sepsis‐related ARDS patients, have poorer recovery from lung injury, lower extubation success rates, higher short‐term mortality, and poor long‐term prognosis.^11^, ^12^ Due to the complex and diverse mechanism of exogenous lung injury in sepsis, the exact mechanism is not fully understood. However, studies have indicated that autophagy plays an important role in the occurrence and development of sepsis‐induced ARDS by regulating the levels of pro‐ and anti‐inflammatory cytokines.^13^, ^14^

In this study, we used RNA‐seq technology to comprehensively analyze the transcriptomic profile of lung tissue in sepsis‐induced ARDS. According to the set screening conditions, we identified 42 autophagy‐related DEGs (26 upregulated and 16 downregulated). Autophagy may play a role in regulating the occurrence and development of inflammation by activating the release of intracellular chemokines, inflammatory mediators, and cytokines through related signaling pathways,^15^ such as JAK‐STAT signaling pathway, HIF‐1 signaling pathway, and toxoplasmosis. In the PPI interaction analysis of 42 differential genes, we identified a total of 42 node proteins, 75 interaction edges, and an average node degree of 3.52, indicating that these proteins have a greater level of interaction than what would be expected for a random set of proteins of the same size and degree distribution drawn from the genome. Such enrichment indicates that the differential proteome may play a biological role in sepsis‐induced ARDS lung injury.^16^ Among the top 10 hub genes, Nod2, Stat3, Hif1a, Hmox1, and Ifng were validated by qRT‐PCR and shown to be clearly associated with autophagy, playing an important role in the biological activity that leads to ARDS lung injury. Further research on these genes is expected to provide a new direction for exploring the mechanism of lung injury.

Under normal circumstances, cell death and survival are in a state of balance. Autophagy can promote cell survival by accelerating cellular metabolism and helping cells adapt to the environment, although this process, in which most of the organelles and cytoplasm in the cell are engulfed, also leads to a form of cell death.^17^ Autophagy is known as type II cell death, whereas apoptosis, which is a type of programmed cell death known as type I cell death.^18^ In this study, GSEA analysis showed that both autophagy‐ and apoptosis‐related genes were upregulated in the ARDS lung injury group (Figure 2C,D) and had significant biological functions. The emergence of autophagy either as a protective role or maladaptive response due to sepsis. It was ensured autophagy to be a protective response. Yet, an overexpression of signal transducers and activators of transcriptions (STATs) in the severe sepsis leads to ALI, describing a maladaptive role. Lung injury can be provoked by ischemia/reperfusion, in which autophagy is stated as the safeguarding mechanism by moderately maintaining the level of Stat3. In normal cells, Stat3 activation by phosphorylation is tightly controlled to avoid abnormal gene expression.^19^ However, Stat3 phosphorylation reached its peak at 15–60 min after stimulation by factors such as cytokines IL‐6, IL‐8, and TNF‐α. Stat3 activation also plays an important role in cell proliferation, survival, invasion, and metastasis, as well as angiogenesis and inflammatory response. Activated Stat3 forms a dimer that enters the nucleus, binds with the downstream Cyclin D1, Cyclin B and cdc2 promoters to initiate transcription, and promote cell cycle progression and cell proliferation.^20^ In addition, activated Stat3 also promotes the survival of injured cells and inhibits apoptosis by regulating the expression of antiapoptotic genes such as *Survivin*, *Bcl‐2*, *Bcl‐xL*, and *Mcl‐1*.^21^

Sepsis‐induced ARDS hypoxic conditions upregulates the transcription of Bcl‐2 interacting protein 3 (BNIP3) by the induction through Hypoxia‐inducing factor 1 (Hif1). Hif1 comprises of two subunits, Hif1α and Hif1β. Hif1α shows unique response to oxygen and expressed during hypoxic conditions while Hif1β, the aryl hydrocarbon receptor‐nuclear translocator, is expressed during normoxic conditions espectively. BNIP3 is a unique member that comes under BH3‐only Bcl‐2 family. It consist two major domains namely transmembrane domain (TM) and PEST domain. Hif1 stabilization acts to express BNIP3 genes and it is known to be a highly regulated process as BNIP3 overexpression may lead to cell death. Cellular stresses developed on exposure to toxic substances like nitric oxide, Lipopolysaccharide, cyanide triggers Hif1‐regulated BNIP3 expression respectively. BNIP3 possesses the ability to provoke cell death via autophagy and apoptosis. Moreover, in the hypoxia and ischemia‐reperfusion conditions induced by septic ARDS, Hif1 plays a protective role in tissue cells by regulating the expression of heme oxygenase 1(Hmox1), and the Hif1a‐Hmox1 axis plays a key role in autophagy induction and inhibition of reactive oxygen species (ROS) production.^22^ Also, it has previously been shown that Hmox1‐generated carbon monoxide (CO) is required for the induction of autophagy and killing of *Mycobacterium tuberculosis* in macrophages in response to immunomodulation by Ifng. Interestingly, Ifng exposure of macrophages induces an increase in intracellular calcium levels that is dependent on Hmox1‐generated CO.^23^ Furthermore, chelation of intracellular calcium inhibits Ifng‐mediated autophagy and mycobacterial clearance from macrophages. These observations suggest that the induction of autophagy is dependent on Hmox1.^24^

Autophagy of alveolar macrophages is a key link in the development of ARDS.^25^ Nucleotide‐binding oligomerization domain 2 (Nod2) functions to monitor bacterial peptidoglycans entering cells and plays an important role in anti‐infection and immune response control.^26^ It is currently believed that the Nod2 receptor is significantly upregulated and promotes autophagy in alveolar macrophages is a component of ARDS pathology. Using a model of lung injury induced by injecting muramyl dipeptide into the airway of mice, Wen et al.^27^ showed that the HMGB1/TLR4 signaling pathway was activated, thereby upregulating Nod2 receptors in alveolar macrophages, and further increasing the expression of autophagy‐related protein Lc3 in alveolar macrophages. Furthermore, Lc3 expression decreased significantly after Nod2 knockout. These findings suggest that upregulation of the Nod2 receptor expression can promote autophagy in alveolar macrophages. These phenomena suggest that the HMGB1/TLR4 signaling pathway activation during lung injury upregulates Nod2 expression to induce autophagy in alveolar macrophages. Further research on the specific mechanism and precise regulation of alveolar macrophage autophagy is expected to become an important target for alleviating ARDS.^28^

## 5 CONCLUSION

In conclusion, we identified 42 potential autophagy‐related genes associated with sepsis‐induced ARDS lung injury through transcriptomic and bioinformatics analyses. Moreover, the six hub genes (Stat3, Il10, Ifng, Hmox1, Hif1a, and Nod2) may affect the development of ARDS by regulating autophagy. These results expanded our understanding of ARDS and might be useful in treatment of exogenous sepsis‐induced ARDS.

## AUTHOR CONTRIBUTIONS

The study conception and design were completed by Xiaomin Li and Yanli Wang. Material preparation, data collection, and analysis were performed by Yongpeng Xie, Wenxia Hu, Xiaobin Chen, Panpan Ren, and Chongchong Ye. The first draft of the manuscript was written by Yongpeng Xie and Jiye Luo, and all authors commented on previous versions of the manuscript. All authors read and approved the final manuscript.

## CONFLICTS OF INTEREST

The authors declare no conflicts of interest.

## ETHICS STATEMENT

This study was approved by the Medical Ethics Committee of Lianyungang Clinical College of Nanjing Medical University (KY20200311001).

## Data Availability

The data used to support the findings of this study are available from the corresponding author upon request.
